# Calcium in Gluten-Free Life: Health-Related and Nutritional Implications

**DOI:** 10.3390/foods5030051

**Published:** 2016-07-15

**Authors:** Urszula Krupa-Kozak, Natalia Drabińska

**Affiliations:** Department of Chemistry and Biodynamics of Food, Institute of Animal Reproduction and Food Research of the Polish Academy of Sciences, Tuwima St., 10, Olsztyn 10-748, Poland; n.drabinska@pan.olsztyn.pl

**Keywords:** calcium supplements, gluten-free diet, coeliac disease, malnutrition, intestinal inflammation, bone alterations, calcium absorption, breadmaking

## Abstract

Calcium deficiency and metabolic bone diseases are a frequent co-morbidity of coeliac disease (CD). Gluten-free diet (GFD) is the only effective treatment of CD. However, CD patients on the strict GFD consume less than the recommended amounts of calcium. In this review, the main etiological factors responsible for calcium deficiency in CD were presented. Additionally, the research on the application of calcium supplements in the gluten-free breadmaking was reviewed, and its effect on the technological and sensory properties of baked products was indicated. Calcium-fortified gluten-free products could increase the calcium content in the diet of CD patients, supplying the amount of calcium they need for prophylactic or therapeutic use. Apart from this, the consumption of the naturally GF products as well as functional ingredients beneficially affecting calcium absorption need to be encouraged.

## 1. Introduction

Calcium is an essential macroelement of the human body. It is mainly deposited in the bones and teeth, where it provides structure and mechanical strength. Nevertheless, Ca is also essential for the proper functionality of the circulatory and neuromuscular systems, acts as a cofactor of several hormones and enzymes, and influences the immunological system. Ca, being a common denominator of bone and intestine, is continuously exchanged between bone and blood which is necessary to maintain extracellular calcium concentrations, regardless of the intake [1]. In food products, Ca occurs mainly in the form of complexes of calcium ions (Ca^2+^) associated with other dietary components, and to be absorbed, it must be released in a soluble and ionised form. Under the physiological conditions, the majority (90%) of dietary calcium is absorbed in the small intestine [2], while the rest is absorbed in the stomach and large intestine.

Nutrition plays an important role in Ca homeostasis; nevertheless, even an adequate dietary Ca intake may not ensure a proper calcium balance. Ca homeostasis is closely controlled with coordinated action of processes such as absorption in the intestine, reabsorption from the kidney and exchange from bones. An adverse calcium balance may result from a poor intestine absorption caused by infection, digestion disease or pathology in the intestine morphology. The group at risk of calcium deficiency includes persons with chronic intestinal diseases, including coeliac disease (CD), which is an immune-mediated systemic disorder triggered by the ingestion of gluten or related prolamines in genetically susceptible individuals [3]. Calcium deficiency and metabolic bone diseases are a frequent co-morbidity in CD patients; approximately 75% of newly diagnosed CD patients have reduced bone mineral density [4], and therefore osteopenia and osteoporosis are considered as signs of atypical CD presentation. In particular, in children and adolescents suffering from CD, Ca deficiency may cause the growth problems and difficulties in peak bone mass achievement [5], whereas in the elderly, Ca deficiency results in a lowered bone mineral density and increased fracture risk [6]. The relationship between CD and bone disorders is well documented [7]; however the pathological bone alterations in CD may be provoked by several factors [8].

Strict and lifelong adherence to a gluten-free diet (GFD) is the only proven treatment for CD [9]. Notwithstanding, several studies have demonstrated that persons suffering from CD may have nutritional deficiencies resulting not only from the intestinal abnormalities, but also from an insufficient supply of nutrients in GFD [10]. Several studies [11,12,13,14] indicated that compared with wheat products, many commercially available gluten-free products provide a low level of vitamins and minerals, including Ca. However, the nutritional status of CD patients depends on the length of time they have lived with active disease, the extent of intestinal damage, the degree of malabsorption, and also on their dietary habits and food choices. GFD can help children and adolescents to recover normal bone mineral density [15]; however, it rarely normalises bone density in adulthood [16]. Currently, some questions concerning the effective treatment of bone problems in CD patients are under debate, including whether GFD alone is sufficient to correct the bone alterations or maybe a supplementation of GFD with calcium and vitamin D needs to be considered for such patients. It is observed that an increasing number of food products are being fortified with Ca (especially dairy products followed by beverages and snacks); however, very few gluten-free products are as Ca-enriched as their gluten-containing counterparts. Calcium-fortified gluten-free products could increase Ca content in coeliac patients’ diet, allowing them to obtain the amount of calcium they need for prophylactic or therapeutic use. The objective of this document is to present the current research on the application of calcium supplements as ingredients of gluten-free products by reviewing the existing data concerning their effect on the technological properties and sensory quality of these products. 

## 2. Aetiology of Calcium Deficiency in CD

### 2.1. Intestine Malabsorption and Inflammation 

In CD, the autoimmune response is mainly targeted at the intestinal mucosa; therefore, when analysing the aetiology of calcium deficiency in CD, the emphasis should be put on the portion of the intestine affected by this disease most severely. CD causes inflammation of the mucosa of the small bowel, from the duodenum to the distal ileum; thus, CD patients generally complain about gastrointestinal problems and frank malabsorption symptoms. Unfortunately, the clinical presentation of CD is highly variable, which complicates its accurate diagnosis. CD can be manifested by a variety of transient or apparently unrelated signs and may affect any organ or tissue. One of its extra-intestinal CD symptoms is bone alternations that are the consequence of impaired calcium absorption, resulting principally from the loss of villous cells in the proximal intestine, where calcium is most actively absorbed [2]. Ca malabsorption is especially observed in acute CD [17,18]. Steatorrhea, alterations in the calcium-transport mechanisms, and vitamin D shortage are the additional factors leading to calcium malabsorption. Similarly to Ca, dietary vitamin D is absorbed through the small intestine as a fat-soluble vitamin along with dietary fat, and is incorporated into chylomicrons. Thus, the primary aetiology of vitamin D deficiency in CD is its malabsorption, with an intestinal mucosal lesion being, however, also a decisive factor for hypovitaminosis D. Calcium absorption in the intestine, reabsorption from the kidney, and exchange from bones is tightly controlled through the coordinated actions of calciotropic hormones, mainly parathyroid hormone (PTH) and 1,25-dihydroxyvitamin D (1,25(OH)_2_D_3_). PTH and vitamin D_3_ exert complex coordinated activities to maintain normal serum calcium levels. Hypocalcemia can induce a compensatory increase of serum levels of PTH, in turn responsible for an increase of bone turnover [19]. Hyperparathyroidism is common in newly diagnosed CD patients (27% in adults; 12%–54% in children) [19,20]. High levels of PTH increase the circulating levels of 1,25(OH)_2_D_3_ by stimulating the renal production of 1-α-hydroxylase, the enzyme responsible for the conversion of 25-hydroxy vitamin D (25(OH)D) to the final hormone 1,25(OH)_2_D_3_. For this reason, increased 1,25(OH)_2_D_3_ levels may be observed in CD [21]. In chronic Ca deficiency, the circulating Ca concentration is maintained largely at the expense of skeletal mass. The cumulative effect of Ca depletion on the skeleton over many years contributes to the increasing frequency of osteoporotic fractures with age [22].

Moreover, a complex role of both local and systemic inflammation in the pathophysiology of bone loss in CD is envisaged [8,23]. In particular TNFα, IL-1 and IL-6 are involved in bone resorption stimulation [24,25]. Additionally, great attention has been given to the RANKL/RANK/osteoprotegerin (OPG) pathway, considered the main signalling system in bone metabolism. Circulating factors secondary to persistent activation of the mucosal immune system could directly interfere with osteoclastogenesis and osteoblast activity. It was shown that in patients following GFD for a mean period of 40 months [26], the prevalence of bone damage is around 40%, and circulating levels of cytokines (IL-6, IL-1beta, TNF-alfa, TNF-beta, IL-12, IL-18, RANK-L, OPG) are significantly lower than in untreated patients, but significantly higher than in healthy volunteers. In CD patients with the recovery of intestinal mucosa, the OPG/RANKL ratio was significantly lower than in healthy controls and positively correlated with low bone mineral density (BMD) [27].

### 2.2. Nutrition

The compliance with a GFD and strict gluten avoidance is not easy as gluten is a ubiquitous food component. Apart from that, gluten-free products are more expensive, and have lower palatability and nutritional value than their gluten-containing counterparts. Dietary surveys have found that CD patients who are on a strict GFD often consume less than the recommended amounts of calcium and vitamin D. Sdepanian et al. [28] confirmed that up to 88% of children and nearly 85% of adolescents with CD adhering to a GFD have inadequate calcium intake. A similarly inadequate Ca intake among children and adolescents on GFD was observed in the study by Blazina et al. [29]. Additionally, Ca supply in the GFD is reduced even more due to a decreased intake of milk and dairy products in an effort to avoid lactose, since secondary lactose intolerance resulting from decreased lactase production by the damaged villi is common among CD patients. 

## 3. Prevention Better than Treatment: Dietary Calcium Sources

### 3.1. Calcium Intake Requirements and Recommendations

**Figure 1:**
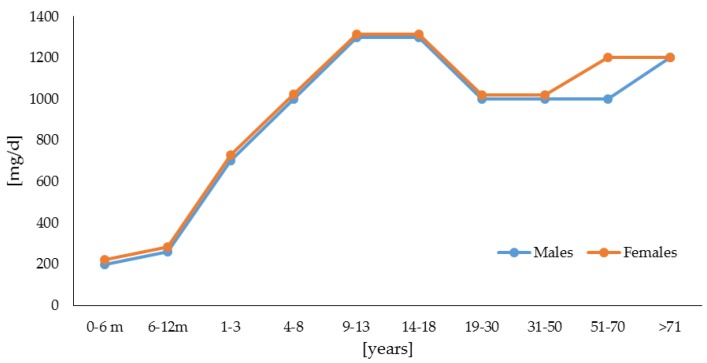
Recommended calcium intake requirements [34].

Literature data indicated a divergent position concerning the GFD as the only therapeutic method of calcium deficiency and bone problems management in patients with CD. Some studies have demonstrated that calcium deficiencies disappear on a strict GFD [30], while other authors have shown that GFD alone does not guarantee adequate calcium intake [11,31,32], and that the CD patients may not be able to meet the recommended daily intake (RDA) level for calcium and vitamin D [33,34]. Calcium is an essential nutrient required in substantial amounts; however, its requirements vary throughout an individual’s life, with greater needs during the periods of rapid growth in childhood and adolescence, and in later life, especially in females (Figure 1).

Unlike other nutrients, the requirement for Ca relates not to the maintenance of the metabolic function of the nutrient but to the maintenance of an optimal reserve and the support of the reserve’s function (i.e., providing internal structural rigidity needed for locomotion and gravity-resisting activity). Therefore, in some conditions, Ca supplementation needs to be introduced. Few studies have analysed the effects of calcium and vitamin D supplementation in CD patients. Meyer et al. [35] observed that bone mass density was not affected by calcium and vitamin D supplements among postmenopausal women, compared to control patients. On the other hand, a long-term calcium (1 g/day) and vitamin D (400 U/day) supplementation increased the bone mineralization in children and adolescents with CD; however, it did not reach the sex- and age-matched values for the control population [36]. Pazianas et al. [37] suggested that the increased calcium intake could potentially compensate for the reduced fractional calcium absorption in treated adult patients with CD. Similarly, Walters [38] recommended a higher-than-the-RDA daily calcium intake for people with CD because of the Ca malabsorption in many patients. Nevertheless, in some special situations, such as severe osteoporosis, it might be useful to begin treatment with hormone replacement therapy (in postmenopausal women) or bisphosphonates [7]; however, there is no systematic data on the efficacy of bisphosphonates or other drugs commonly used for osteoporosis in patients with CD.

### 3.2. Dietary Calcium Sources and Calcium Bioavailability

The major source of Ca in the diet is milk and dairy products, whereas the major non-dairy Ca sources are green leafy vegetables (kale, turnip greens, Chinese cabbage), which provide approximately 7% of dietary Ca [39]. Other excellent sources of Ca are fish, eggs and nuts. However, besides the amount of Ca in the diet, the absorption of dietary Ca in foods is also a critical factor in determining the availability of Ca for bone development and maintenance. Calcium bioavailability depends on its chemical form and factors affecting its solubility. Low pH, basic amino acids, lactose, organic acids, bile salts and an adequate calcium/phosphorus ratio increase calcium bioavailability, whereas higher pH, non-soluble dietary fibre, phytates and oxalates greatly reduce calcium absorption. In general, the bioavailability of Ca is increased in the presence of agents that bind Ca or form insoluble Ca salts. A number of food constituents have been suggested as potential enhancers of Ca absorption. Certain non-digestible oligosaccharides can improve Ca absorption in adolescents and adults [40]. Milk components, such as lactose, lactulose and casein phosphopeptides, have been proposed as potential enhancers of Ca absorption [41]. Additionally, Ca from the animal sources (milk and dairy products) is absorbed more easily than from foods of plant origin [42]. 

In several developed countries, an important source of Ca is the Ca-fortified foods and beverages [43]. Recently, dietary supplements became an important source of dietary Ca. According to Bailey et al. [44], about 43% of the United States population and almost 70% of older females report supplemental calcium use. Nowadays, multiple forms of calcium supplements are commercially available, but several factors, especially medical conditions (impaired gastric acid secretion, and high risk for kidney stone formation, lactose intolerance) need to be taken into account before making the selection of an appropriate Ca supplement. Calcium supplements vary considerably in calcium content, with the largest percent of calcium in calcium carbonate (40%), and with other salts such as citrate, lactate and gluconate constituting 21%, 14% and 9.3% of calcium, respectively [45]. It has been reported that calcium carbonate and citrate are the dominant forms in the calcium supplements used worldwide [46]. Compared to calcium carbonate, calcium citrate is absorbed regardless of gastric acidity; thus, persons producing less gastric acid or receiving drugs that lower acidity in the stomach, such as proton pump inhibitors, H2 blockers, antacids and anticholinergics, may utilise this salt form optimally [47]. Additionally, calcium citrate is better absorbed than calcium as carbonate, causing a greater rise in serum calcium and a greater fall in serum parathyroid hormone (PTH) [48]. Another form of Ca supplement is calcium format, which showed a better ability to deliver Ca to the bloodstream after oral administration than calcium carbonate and calcium citrate [49]. 

## 4. Calcium in Gluten-Free Breadmaking 

### 4.1. Technological Challenge: The Case of Gluten-Free Breadmaking

Although cereals are used extensively in food products, their proteins cannot be tolerated in some individuals. Ingestion of gluten-containing food by susceptible individuals has been associated with gluten-related disorders. The discovery of the relationship between gluten ingestion and the disease emphasised an urgent need for the introduction of a new way of eating habits, consequently leading to the development of GFD. The term “gluten-free” refers to foods containing less than 20 ppm of gluten [50]. 

It is generally accepted that the use of wheat, rye, barley is not allowed in gluten-free breadmaking. Instead, the naturally gluten-free (GF) sources have been used in GF formulations [51,52,53]. The absence of gluten presents a great challenge to both cereal technologists and bakers. In fact, due to its unique properties, gluten is the main structure-forming complex in wheat bread. It has an exceptional ability to form cohesive viscoelastic dough capable of entrapping gas during fermentation and baking and to provide a good crumb structure in the breads obtained [54]. GF dough lacks a natural viscoelastic network, and therefore it is less cohesive and elastic, as well as more sticky and difficult to handle compared to the traditional wheat dough [55]. Moreover, in order to form an acceptable consistency, GF flour requires a higher amount of water than wheat flour [56].

Several studies have been focused on the design of a gluten-free matrix to overcome the negative impact on the network with the absence of gluten [57]. Hydrocolloids (HPMC, guar gum, xanthan gum) [58,59], enzymes (transglutaminase, proteases) [60], proteins from both animal and plant origin (milk protein, egg albumins and soy protein) [61,62,63] and different gluten-free flours (corn, teff, buckwheat, quinoa, sorghum, lupine) and starches (corn, cassava, potato) [64] were used in order to improve the overall quality of gluten-free bakery products. 

### 4.2. Calcium Supplements in Gluten-Free Breadmaking

Enrichment of Ca in wheat flour is mandatory or recommended in many countries, and is a relatively cheap and effective method of Ca dietary supply, taking into account the fact that the consumption of flour and bread is common. In Denmark, flour is fortified with calcium as calcium carbonate, similarly to Great Britain, where the amount of Ca in the form of CaCO_3_ ranges from 900 to 1500 mg of Ca/1 kg of flour [65]. According to the United States and Canadian enrichment standards, flour can be fortified to contain 2.1 g and 1.1 g of Ca/1 kg [66]. Several Ca salts are applied in conventional flour fortification, such as calcium carbonate, tricalcium phosphate, calcium lactate, and calcium citrate malate (CCM). Recently, Reinwald et al. [67] highlighted the important roles that CCM can play during various life stages. Authors demonstrated that CCM facilitates a calcium retention and bone accrual in children and adolescents, and effectively promotes the consolidation and maintenance of bone mass in adults. 

The application of calcium supplements in breadmaking, besides the improvement of the nutritional quality of the final product, which has an evident health effect, may additionally result in some functional benefits, such as the enhancement of bread flavour and texture, and shelf-life extension. An early study by Wang and Tang [68] indicated the improvement of the dough expansion without adverse effects on the quality of calcium lactate–fortified bread. Similarly, Sudha and Leelavathi [69] analysed the influence of calcium salts on rheological characteristics and breadmaking quality of flour, and demonstrated that calcium salts have affected the dough stability and extensibility-to-tenacity ratio. Calcium propionate is widely applied in breadmaking as an antifungal agent. Belz et al. [70] confirmed that the use of calcium propionate (0.3%) prolongs the shelf-life of a low-salt bread up to 12 days. 

In contrast to a conventional wheat flour, Ca fortification of gluten-free flours is uncommon. Moreover, a very limited number of studies have analysed the influence of calcium additives on the characteristics of gluten-free dough as well as on the technological properties and sensory attributes of the gluten-free baked products. In general, the Ca fortification of GF formulations is expected to increase the calcium content in the final bread without a negative effect on its overall quality. According to the Federal Food and Drug Administration (FDA), a good Ca source provides at least 10%–19% of the recommended daily intake (RDI) for calcium, while an excellent source of Ca is considered to provide at least 20% of the RDI of calcium. Krupa-Kozak et al. [71] assessed the influence of different calcium salts (calcium lactate, calcium citrate, calcium chloride and calcium carbonate) on GF dough and bread characteristics and showed that it is possible to obtain a GF bread providing more than 10 mg Ca/g, which according to the Food and Agriculture Organization of the United Nations (FAO)/World Health Organization (WHO) [72] could be considered as an excellent Ca source. Additionally, the experimental GF bread with Ca carbonate obtained in this study was characterised with the highest scores in the sensory evaluation, as its crumb was softer and springier in comparison with the control GF bread without calcium salts. In the same study, the influence of calcium salts on the rheological behaviour of GF dough was also analysed. The authors observed a very low consistency of the control GF dough composed mainly of corn and potato starches in the initial stage of mixing and heating, due to the high hydration and very low protein content. The application of calcium salts considerably influenced the dough’s consistency [71]. Calcium citrate and carbonate increased the dough’s consistency during heating. In contrast, calcium lactate and chloride induced the opposite effect, resulting in delayed corn starch swelling, and calcium chloride lowered the maximum consistency. The changes in the GF dough consistency evoked by calcium supplements may possibly exert an impact on the quality of the GF bread obtained. Matos and Rosell [73] suggested that an increase in the dough consistency during cooling indicates that dough presents a high retrogradation tendency and, consequently, the baked product prepared from this dough would undergo a high staling rate during storage. 

In general, the technological quality of GF bread is low, as it exhibits a crumbling texture, and poor mouth feel, colour and flavour [74]. Although the evaluation of the texture of bread crumbs is necessary for its quality assurance and consumers’ acceptability, the analysis of the textural properties of the GF bread crumb is difficult. This stems from the fact that the distribution of cells within the bread crumb is not homogeneous and dissimilarity in texture properties between the upper and the bottom part of bread slices occurs, being influenced by the differences in the thickness of the cell walls and the moisture distribution [75]. Calcium supplements may favourably influence both the baking characteristics and consumer opinions concerning the appearance and palatability of GF breads. Application of a mixture of two organic calcium supplements, calcium caseinate (low-lactose dairy products of beneficial influence on the sensory quality of gluten-free bread) and calcium citrate (rich in elemental Ca), resulted in GF bread with increased specific volume, fine-coloured crust and pleasant sensory characteristics [76].

### 4.3. Other Calcium Sources in Gluten-Free Breadmaking

Calcium-fortified GF food products could provide additional Ca sources helpful for meeting the dietary requirements; however, the absorption of Ca from foods is a critical factor in determining the availability of Ca for bone development and maintenance. Ca bioavailability depends on its chemical form and factors affecting its solubility. Thus, there is a need to identify the food sources and/or functional food ingredients that may positively influence Ca absorption in order to ensure that Ca bioavailability from foods can be optimised [77]. Moreover, it must be considered that calcium fortification can also affect the bioavailability of other mineral elements such as magnesium.

A number of foods have been suggested as potential enhancers of Ca absorption, including milk components, such as lactose, lactulose and casein phosphopeptides. Therefore, Ca fortification of the GF products by the addition of dairy compounds or alternatively low-/free-lactose products is an important technological approach [61,63,78]. Literature data indicated that also a dietary application of some non-digestible fructooligosaccharides (FOS) can improve Ca absorption and beneficially affect bone mineralisation [79,80,81,82]. Thus, their application in GF foods represents an opportunity of increasing the Ca uptake from the diet. Recently, the study by Krupa-Kozak et al. [83] has demonstrated that inulin-type fructans, especially short-chain FOS, significantly increased Ca uptake from the digestion of the calcium-supplemented GF bread in vitro. Finally, an important approach of GF technology aimed to improve their nutritional quality, including Ca content, involves the use of a wide range of naturally gluten-free grains (oat, sorghum, millet), legumes and pseudo-cereals rich in valuable nutrients, bioactive compounds and minerals, including Ca. Currently, amaranth, quinoa and buckwheat are emerging as healthy alternatives to gluten-containing grains [52,84]. Amaranth [85] and buckwheat [52] are generally a good source of important nutrients and minerals, including Ca; additionally, the pseudo-cereals–containing gluten-free breads showed significantly higher levels of protein, fat, fibre and Ca than the control gluten-free bread.

## 5. Conclusions 

Summarizing, the nutritional deficiencies, including Ca deficiency, in CD result mainly from intestinal abnormalities and Ca malabsorption; however, insufficient Ca supply in GFD is also of great issue. All these factors affect bone alterations; thus, when diagnosing CD, its possible connection with reduced bone mineral density, osteoporosis, and related fractures should always be taken into account. At present, the only effective treatment of CD involves a strict lifelong GFD [86,87]. Ca is an essential nutrient very important for preventing and treating osteoporosis. Ca-fortified GF foods are needed for all CD individuals who do not consume as much Ca as recommended in the dietary guidelines. These are also a great challenge for the GF food industry. In addition, consumption of the natural GF foods as well as foods containing ingredients that may positively influence Ca absorption, such as FOS, need to be encouraged. Moreover, the investigation of increased/corrected calcium intake, the use of Ca supplements and vitamin D metabolites, as well as drugs commonly recommended for primary osteoporosis in CD patients is required.
